# Association between Cognitive Restraint, Uncontrolled Eating, Emotional Eating and BMI and the Amount of Food Wasted in Early Adolescent Girls

**DOI:** 10.3390/nu10091279

**Published:** 2018-09-10

**Authors:** Jinan C. Banna, Chloe E. Panizza, Carol J. Boushey, Edward J. Delp, Eunjung Lim

**Affiliations:** 1 Department of Human Nutrition, Food and Animal Sciences, College of Tropical Agriculture and Human Resources, University of Hawai’i at Manoa, Honolulu, HI 96822, USA; cpanizza@hawaii.edu; 2 University of Hawai’i Cancer Center, Honolulu, HI 96813, USA; cjboushey@cc.hawaii.edu; 3 School of Electrical and Computer Engineering, Purdue University, West Lafayette, IN 47907-2035, USA; ace@ecn.purdue.edu; 4 Department of Complementary & Integrative Medicine, John A. Burns School of Medicine, University of Hawai’i at Manoa, Honolulu, HI 96813, USA; lime@hawaii.edu

**Keywords:** early adolescents, mobile food record, food waste, plate waste, eating behavior, portion size, dietary assessment, uncontrolled eating, cognitive restraint, emotional eating

## Abstract

Understanding of behavioral factors associated with obesity is of importance in addressing this issue. This study examined the association between cognitive restraint, uncontrolled eating, emotional eating and body mass index (BMI) and amount of food plated, consumed, leftovers, and leftover food thrown into the trash (food wasted) in early adolescent girls nine to 13 years in O’ahu, Hawai’i (*n* = 93). Food plated, consumed, leftovers, and food wasted were estimated using a three-day mobile food record™ (mFR). Weight and height were measured to compute BMI (kg/m^2^). The three-factor eating questionnaire provided a score from 0 to 100 for cognitive restraint, uncontrolled eating, and emotional eating. Higher scores are indicative of greater cognitive restraint, uncontrolled eating, and emotional eating. Pearson’s correlations were computed to examine the relationship between three factor eating scores and BMI. General linear models were conducted to examine the effect of each of three-factor eating scores on food plated, consumed, leftovers, and food wasted. Cognitive restraint was positively correlated with BMI (*r* = 0.36, *p <* 0.001) and with BMI *z*-score (*r* = 0.40, *p <* 0.001). There were no associations between three-factor eating scores and food plated, consumed, leftovers, and food wasted at lunch. However, at dinner, total energy plated, left over, and food wasted increased by 4.24 kcal/day (*p* = 0.030), 1.67 kcal/day (*p* = 0.002), and 0.93 kcal/day (*p* = 0.031), respectively, with a unit increase in uncontrolled eating score. Similarly, total energy plated and energy left over at dinner increased by 3.40 kcal/day (*p* = 0.045) and 1.51 kcal/day (*p* = 0.001), respectively, with a unit increase in emotional eating score. Additional research should examine the specific roles of cognitive restraint, uncontrolled eating, emotional eating and food waste in the development of obesity in adolescents.

## 1. Introduction

Rates of childhood obesity in the US are high. The National Health and Nutrition Examination Survey 2011–2014 reported 17.0% of children and adolescents aged 2–19 years were considered obese [1]. In 2015, 13% of high school students in grades 9–12 in Hawaii were obese [2]. This represents an increase over the past few decades among students in Hawaii compared to 10% in 1999 [2]. Numerous factors at the individual, interpersonal, environmental and macrosystem levels contribute to obesity. In determining courses of action to reduce the rates of child obesity, understanding behavioral issues associated with obesity would provide important insight.

One of the tools used to examine behaviors related to development of obesity is the Three-Factor Eating Questionnaire (TFEQ) [3]. The TFEQ provides a score for cognitive restraint (conscious restriction of food in order to control or lose weight), uncontrolled eating (tendency to eat more than usual due to a loss of control over intake accompanied by subjective feelings of hunger), and emotional eating (inability to resist emotional cues) [3]. While several studies have examined these behaviors in adults and their relationship with weight, few have applied the TFEQ to adolescents. Studies conducted with adolescent groups have generally yielded similar results, such as a positive relationship between cognitive restraint and body weight. A study of Turkish adolescents, for example, found body mass index (BMI) was significantly and positively correlated with cognitive restraint and emotional eating [4]. Another study of adolescents in Canada found rigid control (a severe restrictive state), disinhibition (high susceptibility to overeat) and emotional susceptibility to overeat were positively related to BMI z-scores for the entire sample [5]. In a study of French adolescents, those who were obese used cognitive restraint more than the normal-weight adolescents as a strategy for regulating dietary intakes [6]. Similarly, a study of Spanish adolescents found those who were normal weight showed a significantly lower cognitive restraint and higher uncontrolled eating than those who were not normal weight [7].

Of additional interest is the relationship between cognitive restraint, uncontrolled eating, emotional eating and amount of food wasted in early adolescents. Food wasted has a significant negative impact on the natural environment [8] and high financial costs [9]. Furthermore, food wasted may contribute to obesity if adolescents discard foods served as part of programs such as the National School Lunch Program (NSLP) and replace those with foods higher in total energy [10]. In particular, adolescent girls have been shown to waste significant amounts of food, wasting more energy, vegetables, chocolate milk, bread, protein, calcium, iron and vitamin A than boys in the NSLP [11,12,13,14]. There are no known studies reporting the relationship between TFEQ scores and food waste in adolescents. Therefore, gaining a better understanding of behaviors associated with wasting food will allow for development of strategies to mitigate food waste and may contribute to obesity prevention efforts.

The relationship between behaviors assessed using the TFEQ and BMI and amount of food wasted has not been examined in adolescents in Hawai’i, a group warranting examination given current obesity rates and suboptimal dietary habits. The purpose of this secondary data analysis was (1) to examine the association between cognitive restraint, uncontrolled eating, emotional eating and body mass index (BMI) and (2) to examine the association between cognitive restraint, uncontrolled eating, emotional eating and energy (kcal) of food plated, consumed, left over, and wasted in early adolescent girls in Hawai’i.

## 2. Materials and Methods

### 2.1. Materials Study Design

This cross-sectional study was conducted in O’ahu, Hawai’i. Data were collected between February and September 2015. The study was approved by the Institutional Review Board at the University of Hawai’i at Manoa. Detailed methods have been published elsewhere [15] and are described briefly below.

### 2.2. Participants

Girls nine to 13 years of age (*n* = 93) residing in O’ahu, Hawai’i and their caregivers were recruited through posting flyers and giving presentations at local schools, youth centers, libraries, sporting clubs and the University of Hawai’i, as well as through snowballing techniques. Child assent and caregiver consent forms were completed prior to the start of data collection.

### 2.3. Study Protocol

Participants attended two contact sessions. In the first session, participants received instruction for using the mobile food record™ (mFR), an application running on mobile devices previously shown to be feasible and acceptable in youth [16,17,18,19], to collect before and after images of all eating occasions over three days. Parents also completed a demographic data form. The second session focused on reviewing the images, clarifying content of the images and obtaining anthropometric measures from girls. Each participant received $50 in gift cards to a state-wide supermarket chain as an incentive.

### 2.4. Assessment of Food Waste

Each participant was provided with an Apple iPod preloaded with the mFR™ app and two small square colored checkerboard fiducial markers which act as a reference of known dimensions and color to assist with food identification and portion size estimation [19]. Participants were instructed to place a fiducial marker in each image to the lower left of food to be consumed. Participants were instructed to take a before image and an after image of everything they ate or drank excluding water using the mFR™ app over three consecutive days, including one weekend day.

As previously described [19], the second session (one week later) involved a review of images collected with the research dietitian, participant, and parent. A standard interview script was used to clarify the brand, type and ingredients of the foods in the images and the fiducial marker, as well as model cups, plates, bowls and measuring cups, was used to verify the quantity of food plated and any food left over [16,19]. Leftover food was recorded as the total amount of edible food that was plated and left uneaten. The mFR™ does not capture how leftovers are disposed; therefore, during this session participants clarified if any leftover food were thrown into the trash. Food thrown into the trash will be referred to as food wasted.

### 2.5. Anthropometry

Height and weight were collected during the second session using a calibrated scale and stadiometer using a standard protocol [20]. BMI (kg/m^2^) was calculated using height and weight and BMI z-score was calculated according to the Centers for Disease Control and Prevention BMI z-score guidelines for girls 5–19 years [21]. A BMI z-score of −3 or less represented severe thinness, −3 to −2 thinness, −2 to 1 healthy weight, 1 to 2 overweight, and greater than 2 obese [21].

### 2.6. Three-Factor Eating Questionnaire: Cognitive Restraint, Uncontrolled Eating, and Emotional Eating

The Three-Factor Eating Questionnaire-Revised 18 Items (TFEQ-R18) consists of 18 items on a 4-point Likert scale (1 = definitely true, 2 = mostly true, 3 = mostly false, 4 = definitely false). Responses to each of the 18 items are summated into scale scores for cognitive restraint, uncontrolled eating, and emotional eating (see Table 1 for details). Cognitive restraint is composed of six items (e.g., I deliberately take small helpings as a means of controlling my weight) to assess conscious restriction of food intake in order to control body weight or to promote weight loss. Uncontrolled eating is composed of 9 items (e.g., Sometimes when I start eating, I just can’t seem to stop) and assesses the tendency to eat more than usual due to a loss of control over intake accompanied by subjective feelings of hunger. Emotional eating is composed of 3 items (e.g., When I feel anxious, I find myself eating) assessing the inability to resist emotional cues. Higher scores in the respective scales are indicative of greater cognitive restraint, uncontrolled, or emotional eating. The raw scale scores are standardized to a 0–100 scale using the following formula.

- Standardized score = [(raw score-lowest possible raw score)/possible raw score range] × 100
- The reliability of each scale was computed using Cronbach’s alphas. The overall reliability was acceptable (Cronbach’s alpha = 0.82). The Cronbach’s alphas for cognitive restraint, uncontrolled eating, and emotional eating were 0.67, 0.83, and 0.75, respectively.

### 2.7. Amount and Percentage of Food Plated, Consumed, Leftover and Wasted

Analyses were limited to those participants with at least two days of recording. RapidCalc, a data entry program developed by the University of Hawai’i Cancer Center, was used for energy analyses [22,23]. Three separate RapidCalc databases were created for total food plated, food left over and food wasted. These three RapidCalc databases were then replicated and edited to provide data by time of day. Time of day was broken down into four periods: 6–9 a.m., 11–2 p.m., 5–8 p.m. and all other times. These time blocks represented breakfast, lunch, dinner and snacks, respectively [24]. RapidCalc automatically calculated total energy (kcal) per day for each dataset.

Data on total energy (in kcal) from food plated, left over and food wasted at lunch time were exported for further analysis. Food consumed was assumed to be food plated—food leftover. Percentage of energy from food leftover and wasted were calculated as follows:

- Percentage energy left over = (total energy left over/total energy plated) × 100
- Percentage energy wasted = (total energy wasted/total energy plated) × 100

### 2.8. Sample Size and Power Calculation

Power calculations were performed on the association between three food behaviors and BMI, based on a previous study [5]. Eighty-two participants were needed to detect statistically significant correlation of 0.3 between BMI and the three food behaviors, with 80% power assuming two-sided significance level of 0.05. This study was not powered to evaluate association between three food behaviors and food plated, consumed, leftovers, and food wasted.

### 2.9. Statistical Methods

Demographic variables were summarized using descriptive statistics such as mean and percentage. Pearson’s correlation coefficients were calculated to measure the association between cognitive restraint, uncontrolled eating, emotional eating and BMI. We also computed partial correlation adjusting for age. To evaluate the effect of each eating factor assessed using the questionnaire, we conducted general linear models on the amount of food plated, consumed, leftover, and wasted adjusting for BMI z group and age. BMI z group was categorized as obese/overweight (i.e., BMI *z*-score > 1) vs. normal/underweight (i.e., BMI *z*-score ≤ 1). All statistical analyses were conducted in SAS version 9.4 and *p*-value < 0.05 was considered statistically significant.

## 3. Results

All 93 participants completed the study. Among them, nine participants did not meet the acceptable criteria of at least 2 days of recording or did not answer any of items on the TFEQ-R18. Consequently, their data were removed from the final analysis and the final sample size was 84 participants.

Table 2 presents descriptive statistics. The mean age was 10.8 years (SD = 1.3) and 48 (57%) girls were Asian. Sixty-seven (83%) mothers recorded a total household income of $60,000 USD or greater and 49 (58%) mothers had at least attended and/or completed graduate school.

Table 3 shows descriptive statistics and correlations between cognitive restraint, uncontrolled eating, emotional eating and BMI. The means of cognitive restraint, uncontrolled eating, and emotional eating were 34.3 (SD = 17.2), 41.5 (SD = 18.6), and 22.9 (SD = 21.4), respectively. There was a significant correlation between cognitive restraint and BMI. Cognitive restraint had a positive correlation with BMI (*r* = 0.36, *p* < 0.001) and with BMI *z*-score (*r* = 0.40, *p* < 0.001). When adjusting for age, significant associations were found between emotional eating and BMI (partial *r* = 0.26, *p* = 0.018) and BMI *z*-score (partial *r* = 0.22, *p* = 0.043) and between uncontrolled eating and BMI (partial *r* = 0.22, *p* = 0.047).

Table 4 presents results from the general linear models testing the association between energy from food plated, consumed, leftover and wasted with each three factor eating score. After adjusting for age and BMI *z*-score group, energy from snack food discarded into the trash increased by an average of 0.51 kcal/day (*p* = 0.021) for every unit increase in cognitive restraint score. Total energy of the plated breakfast decreased by an average of −1.63 kcal/day (*p* = 0.044) for every one unit increase in cognitive restraint score. However, total energy plated, left over, and food wasted at dinner increased by an average of 4.24 kcal/day (*p* = 0.030), 1.67 kcal/day (*p* = 0.002), and 0.93 kcal/day (*p* = 0.031), respectively, for every one unit increase in uncontrolled eating score. Similarly, the total energy plated and energy leftover at dinner increased by an average of 3.40 kcal/day (*p* = 0.045) and 1.51 kcal/day (*p* = 0.001), respectively, with every one unit increase in emotional eating score. In addition, the percentage of energy leftover at dinner increased by 0.11% (*p* = 0.034) with every one unit increase in emotional eating score.

## 4. Discussion

Among adolescent girls in Hawai’i, there was a positive correlation between cognitive restraint and BMI. There was also a significant partial correlation between BMI and emotional eating, and BMI *z*-score and emotional eating.

The positive correlation revealed between restrained eating and BMI aligned with results of previous studies. In a study of French adolescents, for example, dietary restraint was positively correlated with overweight [25]. However, in the current study, there was no correlation between energy (kcal) intake and restrained eating, while previous studies revealed a positive correlation [26,27]. This finding in previous studies may be explained by the overeating that may result from dietary restraint, leading to a cycle of weight gain and restriction and unsuccessful restraint that fosters storing of excess energy. Those who are overweight or obese may also be more likely to be on a diet and restricting intake for weight loss. In the current study, there may be other factors that explain the positive correlation between restrained eating and BMI. For example, the responses to the items on restrained eating may not be an accurate reflection of behavior if overweight and obese participants considered restrained eating to be a socially acceptable means of controlling weight and responded accordingly regardless of whether they were actually behaving in this manner.

There was also a significant partial correlation found between uncontrolled eating and emotional eating and BMI. Previous studies have also revealed a positive relationship between these factors and weight [5,27]. A study of Dutch adolescents, for example, revealed that overweight children had higher disinhibition scores [27]. Similarly, a study of Spanish youth demonstrated that overweight participants scored higher on external eating, which involves a decreased sensibility to internal signals of hunger and satiety, compared to normal weight children [28]. Other studies, in contrast, have found a negative relationship, with lower uncontrolled eating scores in youth with higher BMI [7]. Overweight or obese children may not answer questions about uncontrolled eating honestly in such studies.

In the current study, uncontrolled eating or emotional eating was also positively associated with total plated and energy leftover at dinner. However, energy wasted at dinner was not correlated with emotional eating; therefore, the leftover food at dinner may have been eaten by someone else or stored for later consumption. In addition, there was a negative association between total plated at breakfast and uncontrolled eating. If adolescents consumed less food at breakfast, they may have had less control at dinner and potentially could have engaged in binge eating behavior.

Except for snacks, cognitive restraint, emotional eating, uncontrolled eating were not associated with food thrown into the trash (energy wasted). For snacks, there was 0.5 kcal of food wasted per 1 unit increase in cognitive restraint score. Thus, from an environmental standpoint, this relationship may not be of importance.

Food plated, consumed, leftover and wasted at lunch were not related to TFEQ scores. Therefore, factors other than cognitive restraint, emotional eating and uncontrolled eating may be contributing to the large amount of lunch time plate waste being discarded in the school environment in Hawai’i [15].

The current study has several limitations. Given the sampling technique used, results may not be generalized to adolescents beyond those who participated. In addition, this is a cross-sectional study, and is not enough evidence to establish a cause and effect relationship between eating behaviors and BMI without further research. Finally, information on food/beverages served and waste were collected using a self-initiated method, thus the possibility exists some food/beverages served may not have been captured using images. On the other hand, eating occasions captured required nothing more than taking a picture. Thus, the content of the images used were likely more descriptive and contextual than eating occasions recorded on paper or recalled by memory.

## 5. Conclusions

Among adolescent girls in Hawai’i, there was a positive correlation between cognitive restraint and BMI, as well as a positive correlation between both uncontrolled eating and emotional eating and food leftover at dinner. Additional research is needed to examine the specific roles of these behaviors in development of obesity in adolescents.

## Figures and Tables

**Table 1:** Three-Factor Eating Questionnaire-Revised 18 Item.

Item	Question	Scale
1	When I smell a sizzling steak or juicy piece of meat, I find it very difficult to keep from eating, even if I have just finished a meal.	UE
2	I deliberately take small helpings as a means of controlling my weight.	CG
3	When I feel anxious, I find myself eating.	EE
4	Sometimes when I start eating, I just can’t seem to stop.	UE
5	Being with someone who is eating often makes me hungry enough to eat also.	UE
6	When I feel blue, I often overeat.	EE
7	When I see a real delicacy, I often get so hungry that I have to eat right away.	UE
8	I get so hungry that my stomach often seems like a bottomless pit.	UE
9	I am always hungry so it is hard for me to stop eating before I finish the food on my plate.	UE
10	When I feel lonely, I console myself by eating.	EE
11	I consciously hold back at meals in order not to weight gain.	CG
12	I do not eat some foods because they make me fat.	CG
13	I am always hungry enough to eat at any time.	UE
14	How often do you feel hungry?	UE
15	How frequently do you avoid “stocking up” on tempting foods?	CG
16	How likely are you to consciously eat less than you want?	CG
17	Do you go on eating binges though you are not hungry?	UE
18	On a scale of 1 to 8, where 1 means no restraint in eating (eating whatever you want, whenever you want it) and 8 means total restraint (constantly limiting food intake and never “giving in”), what number would you give yourself? *	CG

CG = Cognitive Restraint Scale; UE = Uncontrolled Eating Scale; EE = Emotional Eating Scale. * The 1–2 scores were coded 1; 3–4 scores were coded 2; 5–6 scores were coded 3; 7–8 scores were coded 4.

**Table 2:** Characteristics of Final Sample (*n* = 84).

**Continuous Variables**	**Mean ± SD**
Age,	10.8 ± 1.3
BMI *z*-score	0.1 ± 1.1
**Categorical Variables**	***n* (%)**
Age category	
9–10 years	35 (42%)
11–13 years	49 (58%)
Race	
White	27 (32%)
Asian	48 (57%)
Other ^a^	9 (11%)
Total household income	
$0–$59,999	14 (17%)
$60,000 or more	67 (83%)
Mother’s education level	
Graduated from a four-year college or university or less	35 (42%)
Attended and/or completed graduate school or more	49 (58%)
Body weight status	
>1 (Overweight or Obese)	16 (19%)
≤1 (Normal or Underweight)	68 (81%)

^a^ Other race includes Native Hawaiian or Other Pacific Islander, American Indian or Alaska Native, Black or African American and Some Other Race [24].

**Table 3:** Correlation of Cognitive Restraint, Uncontrolled Eating, and Emotional Eating Score with BMI (*n* = 84).

		Three Factor Eating Questionnaire, Correlation (Partial Correlation)		
Variable	Mean ± SD	Cognitive Restraint	Uncontrolled Eating	Emotional Eating
BMI	18.9 ± 4.0	0.36 *** (0.41 ***)	0.13 (0.22 *)	0.19 + (0.26 *)
BMI *z* score	0.1 ± 1.1	0.40 *** (0.41 ***)	0.11 (0.14)	0.20 + (0.22 *)

+ *p* < 0.10; * *p* < 0.05; *** *p* < 0.001. Partial correlation was computed adjusting for age.

**Table 4:** Association between Cognitive Restraint, Uncontrolled Eating, and Emotional Eating Score and Amount of Energy Plated, Consumed, Leftover, and Wasted.

	Cognitive Restraint			Uncontrolled Eating			Emotional Eating		
Response	B	SE	*p*-Value	B	SE	*p*-Value	B	SE	*p*-Value
***Whole Day***									
Total Plated (kcal/day)	1.40	2.79	0.616	4.61	2.57	0.077	2.87	2.25	0.206
Total Consumed (kcal/day)	1.13	2.61	0.667	2.88	2.43	0.239	1.69	2.12	0.429
Leftover Food (kcal/day)	0.28	1.12	0.806	1.73	1.04	0.101	1.19	0.91	0.194
Food Wasted (kcal/day)	−0.38	0.77	0.623	0.89	0.72	0.219	−0.22	0.63	0.722
Food Wasted (%) *^a^*	−0.01	0.04	0.750	0.00	0.04	0.917	−0.05	0.03	0.167
Leftover Food (%) *^b^*	−0.01	0.06	0.911	0.03	0.06	0.574	0.03	0.05	0.527
***Breakfast***									
Total Plated (kcal/day)	−0.36	0.87	0.679	***−1.63***	***0.80***	***0.044***	−1.00	0.70	0.156
Total Consumed (kcal/day)	−0.36	0.79	0.649	−1.33	0.73	0.074	−0.63	0.64	0.330
Leftover Food (kcal/day)	0.00	0.36	0.994	−0.30	0.33	0.368	−0.37	0.29	0.204
Food Wasted (kcal/day)	−0.23	0.18	0.200	0.00	0.17	0.979	−0.24	0.15	0.107
Food Wasted (%) *^a^*	−0.03	0.04	0.449	0.03	0.04	0.464	−0.02	0.03	0.630
Leftover Food (%) *^b^*	−0.07	0.06	0.257	−0.01	0.05	0.787	−0.05	0.05	0.279
***Lunch***									
Total Plated (kcal/day)	−0.04	1.45	0.979	0.01	1.36	0.996	−0.34	1.18	0.771
Total Consumed (kcal/day)	0.35	1.34	0.793	−0.26	1.26	0.837	−0.17	1.09	0.876
Leftover Food (kcal/day)	−0.39	0.44	0.380	0.27	0.42	0.526	−0.17	0.36	0.634
Food Wasted (kcal/day)	−0.64	0.39	0.105	0.06	0.37	0.877	−0.50	0.32	0.117
Food Wasted (%) *^a^*	−0.04	0.06	0.538	−0.04	0.06	0.506	−0.09	0.05	0.069
Leftover Food (%) *^b^*	0.02	0.07	0.778	−0.03	0.07	0.656	−0.05	0.06	0.415
***Dinner***									
Total Plated (kcal/day)	−0.39	2.10	0.855	***4.24***	***1.92***	***0.030***	***3.40***	***1.67***	***0.045***
Total Consumed (kcal/day)	−0.84	1.84	0.650	2.56	1.71	0.138	1.89	1.49	0.209
Leftover Food (kcal/day)	0.45	0.59	0.443	***1.67***	***0.52***	***0.002***	***1.51***	***0.45***	***0.001***
Food Wasted (kcal/day)	−0.02	0.46	0.964	***0.93***	***0.42***	***0.031***	0.47	0.37	0.212
Food Wasted (%) *^a^*	0.00	0.05	0.934	0.04	0.04	0.345	−0.01	0.04	0.856
Leftover Food (%) *^b^*	−0.01	0.07	0.914	0.07	0.06	0.242	***0.11***	***0.05***	***0.034***
***Snack***									
Total Plated (kcal/day)	2.14	1.96	0.278	1.99	1.84	0.282	0.87	1.60	0.587
Total Consumed (kcal/day)	1.98	1.76	0.264	1.91	1.65	0.251	0.60	1.44	0.676
Leftover Food (kcal/day)	0.16	0.49	0.740	0.09	0.46	0.852	0.27	0.40	0.496
Food Wasted (kcal/day)	***0.51***	***0.22***	***0.021***	−0.09	0.21	0.658	0.05	0.18	0.793
Food Wasted (%) *^a^*	0.06	0.03	0.057	−0.03	0.03	0.264	0.00	0.03	0.914
Leftover Food (%) *^b^*	0.04	0.06	0.443	0.02	0.05	0.775	0.03	0.05	0.504

B = parameter estimate. SE = Standard error. *^a^* Percentage food wasted = (total food wasted/total food plated) × 100. *^b^* Percentage food left over = (total food left over/total food plated) × 100. General linear model was conducted on each row variable as a dependent variable and each column variable as an independent variable, controlling for age and BMI *z*-score group (categorized as BMI *z*-score > 1 vs. BMI *z*-score ≤ 1). Bold italic indicates that the column factor eating questionnaire is *p*-value < 0.05.
